# Production and Physicochemical Properties of Starch Isolated from Djulis (*Chenopodium formosanum*)

**DOI:** 10.3390/foods8110551

**Published:** 2019-11-05

**Authors:** Wen-Chien Lu, Yung-Jia Chan, Fang-Yu Tseng, Po-Yuan Chiang, Po-Hsien Li

**Affiliations:** 1 Department of Food and Beverage Management, Chung-Jen Junior College of Nursing, Health Sciences and Management, No. 217, Hung-Mao-Pi, Chia-Yi City 60077, Taiwan; 2 College of Biotechnology and Bioresources, Da-Yeh University, No. 168, University Rd., Dacun, Chang-Hua 51591, Taiwan; 3 Department of Medicinal Botanical and Health Applications, Da-Yeh University, No. 168, University Rd., Dacun, Changhua 51591, Taiwan; 4 Department of Food Science and Biotechnology, National Chung-Hsing University, Taichung 402, Taiwan

**Keywords:** djulis (*Chenopodium formosanum*), starch isolation, crystallinity, morphology, pasting properties

## Abstract

Djulis (*Chenopodium formosanum* Koidz.) is an annual fast-growing underutilized pseudo cereal with a high percentage of starch content. In this study, djulis starch was extracted from the flour of dried grains by three different isolation procedures: (1) hydrochloric acid (HCl) isolation procedure (HP); (2) deionized water isolation procedure (WP); and (3) sodium hydroxide (NaOH) isolation procedure (NP), followed by investigation of the physicochemical properties of the isolated djulis starch. The amylose content of HP, WP, and NP was 22.14%, 24.15%, and 22.43%, respectively. For scanning electron microscopy (SEM) morphological observation, djulis starch presented a polygonal shape with granule sizes of 0.56–1.96, 0.74–3.02, and 0.62–2.48 μm, respectively. Djulis starch showed the classification of typical A-type x-ray patterns, and the relative degree of crystallinity for HP, WP, and NP was 33.15%, 36.17%, and 37.42%, respectively. Differential scanning calorimetry (DSC) analysis was used to determine the transition temperatures, transition range, and enthalpies of the gelatinization of starches. HP and WP isolated starch exhibited the highest ΔH 9.24 and 8.51 J/g, respectively, whereas NP starch showed the lowest ΔH of 6.95 J/g. The pasting temperatures of HP, WP, and NP isolated starch, which were analyzed by using a Rapid Visco Analyzer (RVA), were 71.70 °C, 72.80 °C, and 69.53 °C, respectively. The dependence of swelling power for the three isolated starches on temperature was tested at 10 °C with intervals between 60 °C and 90 °C. In short, the NP isolation procedure with a stable reaction is compelling from a technological point of view.

## 1. Introduction

Djulis (*Chenopodium formosanum* Koidz.) belongs to the class *Dicotyledoneae*, a genus of *Chenopodium* that consists of more than 100 species of perennial and annual herbaceous flowering plants, is a native pseudo-cereal grain native grown in Taiwan [1]. Djulis is a traditional crop that has been typically cultivated by Taiwan’s aborigines in some aboriginal inhabited areas for more than 100 years. Djulis is an important grain crop for human and animal foodstuff because of its high nutrient content such as functional dietary fiber, starch, protein, and a balanced amino acid spectrum with high lysine and methionine contents [2]. In recent years, many researchers have been attracted to djulis due to its attractive color, functional and nutritional compositions, and bioactive compounds [3]. Furthermore, focus on the products of djulis has significantly increased to further develop enriched functional foods [4]. 

Starches are semi-crystalline polymer forms that are primarily composed of amylose and amylopectin. Amylose is characterized by long linear chains of α-(1→ 4) glycosidic linkages with relatively few α-(1→ 6) linked branches, while amylopectin is highly branched molecules of shorter α-(1→ 4) glycosidic linkage glucose molecules and more frequently α-(1→ 4) branches [5,6]. The major sources of starch, for example, cereal crops and root vegetables, have been extensively reported and the physicochemical properties have been discussed in previous studies [7,8].

The physicochemical properties of starch including its morphological features, the characterization of the starch, amylose capacity, crystallization and thermal properties, swelling indexes, and pasting features establish the utilization variety of starch in food and non-food industries [9]. Approximately 3000 million tons of starch is extracted per year globally from distinct types of cereals, grains, and root crops, of which 60% is utilized in food processing such as mayonnaise, baby food, bakery products, beverages, meat commodity, fat substitutes, and confectionery, and 40% is used in pharmaceuticals, for example, tablets and dusting powder, and non-edible products (agriculture, paper, textile, and plastic) [10].

Starch isolated from different genotypes and botanical sources with suitable characteristics is gaining more attention in the food industry because it does not require chemical or genetic modification [11,12]. Therefore, the morphology and physicochemical properties of new types of starches must be investigated to look over its potential applications, while exploring their most suitable usability as an additive for food or non-food industrial purposes. Furthermore, the effects of isolated methods on starch must be well characterized as only a few studies have been carried out on the starch in djulis grains.

Starch can be isolated from native sources in several ways including alkali treatment, deionized water treatment, and enzyme digestion [13,14]. For starch separated from rice flour, alkali treatment produces a starch with a higher peak paste and final viscosity that swells more rapidly than starch isolated by other methods [15]. Picking a suitable starch-extracting method is important to acquire a purified product with the optimum yield and recovery, but at the least cost, and the minimal amount of sewerage as possible. A series of interrelated stages is needed to allow the non-starchy fraction to be eliminated, not beyond arousing the effect of the granule original structure and minimally associated toxicities [16,17].

Isolation methods intensely manipulate different starch extension characteristics. Hence, this study examined a suitable process to isolate starch from djulis grains. A hydrochloric acid treatment procedure (HP), deionized water treatment procedure (WP), and alkali treatment procedure (NP) was verified for producing a higher yield and recovery of djulis starch. Moreover, the effects of starch isolation procedures toward the chemical compositions and morphology, together with the crystalline, thermal, and pasting properties of starch granules, have been reported.

## 2. Results and Discussion

### 2.1. Chemical Composition, Starch Yield, and Recovery

The chemical composition of djulis starch isolated by the three procedures is presented in Table 1 and Table 2. The djulis starch contained 0.22–0.37% protein, 4.89–6.35% moisture, 0.75–0.84% lipids, and 0.11–0.14% ash. The amylose content affects the abundance of physical properties of the starch, for instance, the temperature of pasting and gelatinization, the efficiency to trap water, crystalline structure, and digestibility [18]. The amylose content from the three treatments varied from 22.14 to 24.15%, with no significant differences between each treatment. In previous reports, the amylose content of *Chenopodium* spp. (*Chenopodium quinoa*) starch was testified to be much lower, which could be attributed to the different botanical species used, cultural conditions, and methods used for starch isolation [19]. Furthermore, Lorentz [20] clarified that the amylose content of *Chenopodium* spp. ranged from 9 to 11%. The damaged starch content with the three isolation procedures ranged from 2.12 to 3.93% (Table 1), as compared to chestnut starch extracted by alkaline and enzymatic treatments, which was found to have a high level of mutilation [17], indicating that only slight destruction to the starch granules was caused by the separation methods. The efficiency of the extracting methods for *Chenopodium* starch was assessed in terms of the yield and recovery. Table 2 shows the yields and recovery of djulis starch isolated by the three different treatments. The starch yield and recovery were higher for WP than HP and NP, which were 30.19% and 79.68%, respectively.

### 2.2. Morphological Characteristics

**Figure 1:**
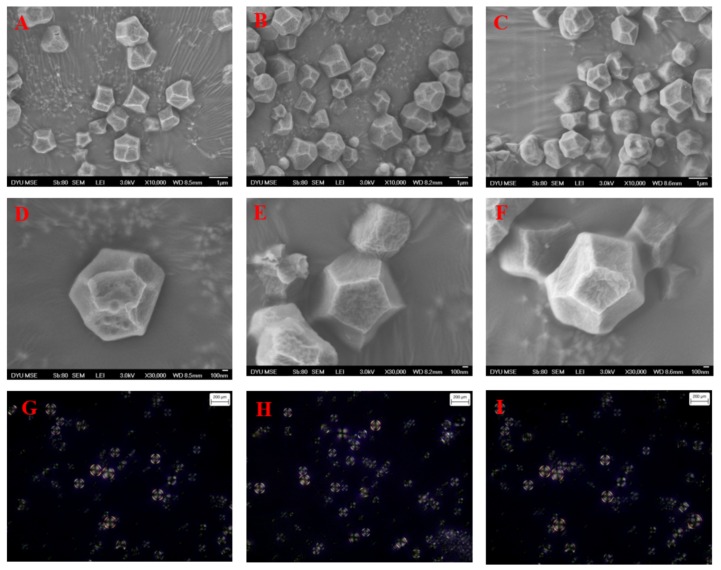
Scanning electron microscopy (**A**–**F**) and polarized light microscopy (**G**–**I**) of djulis starch by the three treatment procedures. Hydrochloric acid isolation procedure (HP) (**A**,**D**,**G**); Deionized water isolation procedure (WP) (**B**,**E**,**H**); and the sodium hydroxide isolation procedure (NP) (**C**,**F**,**I**).

For SEM morphological observation, the starch granules isolated by the three different methods exhibited a polygonal and angular shape (Figure 1A–F). The size of the starch granules differed with a mean size of 1.57, 1.84, and 1.62 μm for HP, WP, and NP, respectively (Table 2). The smaller djulis starch granules found in nature are grouped in clusters, which provide unique properties. The granules’ size and shape of one and the other samples was observed to agree with that described in the same species (*Chenopodium quinoa*) in previous research [21]. Figure 1G–I illustrates the polarized light micrographs (PLM) of djulis starch isolated by the three various isolation procedures. The granules of starch had an unbroken structure, expressing a well-defined birefringence pattern with a dark cross. Birefringence only indicates a high degree of molecular orientation within the granule and does not refer to any exceptional crystalline form. The results obtained corresponded to the studies of [22] toward the *Chenopodium* spp., which showed spherical and polygonal isolated quinoa starch with a submicron size. Djulis starch did not exhibit the cumuli conformation after the different starch isolation procedures. This conformation, in addition to the size and shape of the granules might affect the physicochemical properties of the samples. In short, the SEM images displayed that when the starch was in the djulis seed, it shaped clusters that were engrossed in a matrix formed by starch granules rich in amylopectin. Similarly, when the starch was separated, the starch formed was spherical and polygonal starch; both with almost the same size range were observed.

### 2.3. Crystalline Properties

**Figure 2:**
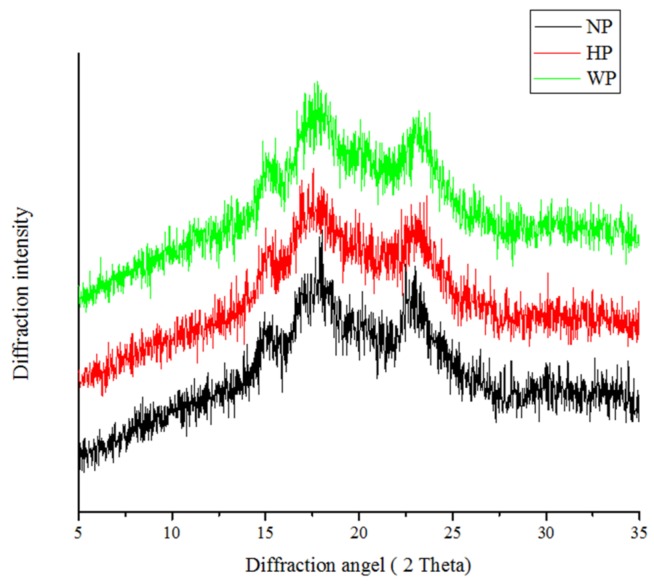
X-ray diffraction patterns of djulis starch by the (1) hydrochloric acid isolation procedure (HP); (2) deionized water isolation procedure (WP); and (3) sodium hydroxide isolation procedure (NP) for djulis starch.

Starch, a semi-crystalline material, could be acknowledged by characteristic x-ray diffraction patterns. The x-ray diffraction patterns of djulis starch is shown in Figure 2. Djulis starch isolated by the three procedures showed an A-type diffraction pattern, with peak intensities at 15.13°, 17.21°, and 23.12° 2θ, respectively. A-type starch is discriminative of greater starches of cereal origin and has intense diffraction peaks at 15° and 23° 2θ, and an uncertain doublet at about 17° and 18° 2θ [23]. The observations agreed with other reports of *Chenopodium* species starch [21], which concluded that the broad peaks established in the x-ray patterns of isolated quinoa starch specified that amylose and amylopectin are composed of nanocrystals [22].

The relative degree of crystallinity of djulis starch was 36.17% (WP), 37.42% (NP), and 35.25% (HP), respectively. The results are comparable with the study by Jan et al. [24] on *Chenopodium* starch. Regarding the crystallinity, previous studies have demonstrated that *Chenopodium* spp. is considered to be a semi-crystalline material due to its x-ray diffraction pattern, which expressed a broad peak, and the reports defined that this was due to the transposition of amorphous and crystalline layers from amylose and amylopectin that form the granule [25]. Nevertheless, Londoño-Restrepo et al. [18] clarified that the broad peaks in the x-ray diffraction pattern could relate to the presence of nano-crystals and lamellae, instead of low crystalline quality. The crystal size, quantity of crystalline regions, amylose chain, and positioning of the double helices within the crystalline areas are crucial to conclude the starch crystallinity, amorphous region, and degree of interaction involving double helices [26].

The amylose content has a significant impact on the crystallinity of starch. The relative degree of crystallinity affects numerous practical properties and biochemical specifications of starch [27]. However, other specifications, for instance, the size of the crystal, number of crystalline regions, positioning of the double helices within the crystalline areas, and the expansion of interaction between double helices are crucial to conclude the crystalline degree [26]. The starch crystallinity differs with the size of the crystal and amount of crystalline region, while the amylose chain is in charge of the amorphous region and orientation of double helices within the crystalline domain with a degree of interaction involving double helices [27].

### 2.4. Swelling Power

**Figure 3:**
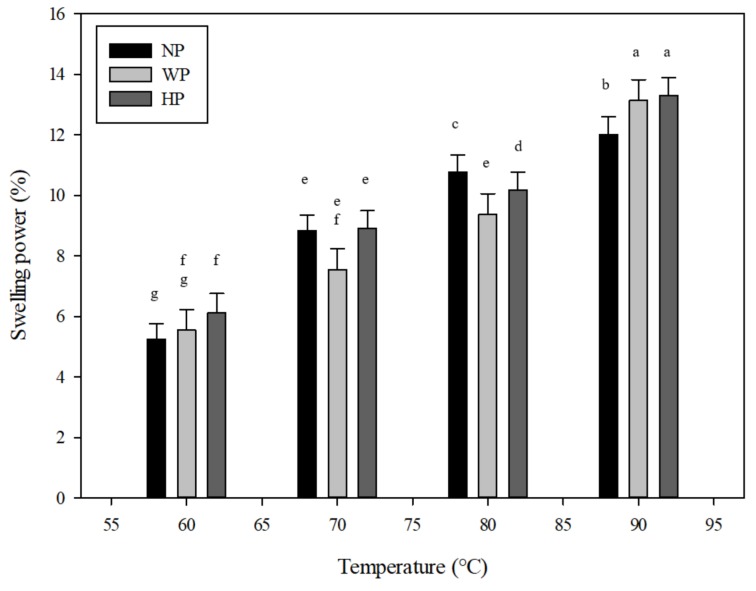
Swelling power of djulis starch by the (1) hydrochloric acid isolation procedure (HP); (2) deionized water isolation procedure (WP); and (3) sodium hydroxide isolation procedure (NP) for djulis starch. Means with the same letters in a column do not differ significantly (*p* < 0.05) (*n* = 3).

The swelling power of djulis starch extracted by the three different isolation procedures was measured at 10 intervals ranging in temperature from 60 °C to 90 °C (Figure 3). The results demonstrated that the swelling power rose gradually with increasing temperature from 60 °C to 90 °C. A rapid increase in swelling power was observed between the temperatures of 70 °C to 90 °C. The ultimate swelling power was detected at 90 °C. HP treatment samples had a higher swelling power at 60 °C when compared to NP and WP. The swelling power for NP and HP isolation starches at 90 °C was 12.02% and 13.31%, respectively. These values approached those discovered for *C. quinoa* starch [20]. The increasing trend of swelling power toward the temperature may be due to the low content of amylose present in the djulis starch samples. As described by Tester and Morrison [28], amylose leads to the solubility of starch while amylopectin primarily affects the starch swelling power. The swelling power of typical starch granules is principally an attribute of amylopectin molecules, with amylose substituting in a role as a dilute [28] or suppressing the strength of amylopectin swelling [29]. The variances in swelling may be accredited to the exquisite structure of the amylopectin molecules due to a lack of significant differences in the amylose content.

### 2.5. Gelatinization Thermal Properties

The djulis starch gelatinization was measured and documented the amount of heat involved by using DSC. The gelatinization parameters of starch are connected to a variety of circumstances as well as the size, proportion, and kind of crystalline organization, the shape of the starch granules as well as the architecture, size, and phosphate groups [30]. Thermal transition temperatures (T_0_, T_p_, and T_c_) in conjunction with an enthalpy of gelatinization (ΔH) and the gelatinization temperature range (ΔT) are summarized in Table 3. The thermal properties (T_0_, T_p_, and T_c_) of starch extracted by the three different isolation methods indicated non-significant differences, which make clear the scarce or null influence of the isolating procedures. The values found for djulis starch were: T_0_ = 59.45 ± 0.47 °C, T_p_ = 64.35 ± 0.62 °C, T_c_ = 72.19 ± 1.73 °C (HP), T_0_ = 60.27 ± 0.54 °C, T_p_ = 65.21 ± 0.22 °C, T_c_ = 72.95 ± 0.38 °C (WP), and T_0_ = 60.73 ± 0.89 °C, T_p_ = 66.02 ± 1.10 °C, T_c_ = 70.74 ± 1.35 °C (NP), respectively. Gelatinization is defined as the disruption of granules, double helical, and loss of crystalline structure of starch [31]. These values were higher when compared to the previous studies about *Chenopodium* spp. with the values confirmed as T_0_ from 44.7 to 57.4 °C, T_p_ from 50.7 to 61.9 °C, and T_c_ from 66.2 to 68.5 °C [30]. Nonetheless, even though the scientific studies were carried out on the same family belonging to *Chenopodiaceae*, nevertheless, the results were incomparable with the existing literature due to the different species.

Starch granules are initiated to swell and the inter-molecular hydrogen bonds of the starch molecules break down in the existence of adequate water and upraised temperature. At greater temperatures, the granule is obstructed entirely, and gelatinization is completed [32]. The HP and WP isolated starch exhibited the highest ΔH 9.24 and 8.51 J/g, respectively, whereas the NP starch showed the lowest ΔH of 6.95 J/g. ΔH demonstrates the complete crystallinity of amylopectin, defined as the number of starch crystallites [28]. The low ΔH values of NP starch are related to its low crystallinity degree.

The ΔT was high for starch obtained by HP (12.74 ± 0.44 °C) and WP (12.68 ± 0.31 °C) when compared with NP (10.01 ± 0.25 °C). Taking into account that thermal properties are closely interrelated with the destabilizing effect of the amorphous zones (branched regions) that sponge up water before the crystalline zones (amylopectin magnificent zone). Thus, the attendance of alkali (NaOH) used in NP treatments may disturb the crystalline domains, triggering the starch to become more amorphous, consequently promoting the melting of the crystallites in a narrow range of temperature [14]. The gelatinization parameters of starch are related to a variety of circumstances as well as the size, proportion, and kind of crystalline organization, the shape of the starch granules, and the architecture, size, and phosphate groups [30].

### 2.6. Pasting Properties

**Figure 4:**
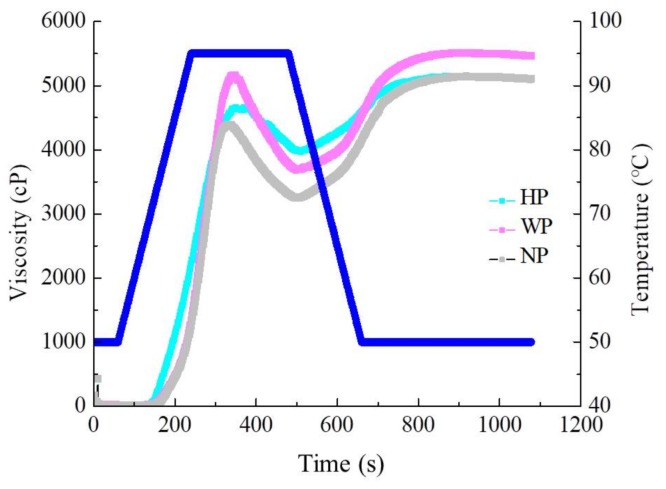
Rapid Visco Analyzer (RVA) viscoamylogram of djulis starch by the (1) hydrochloric acid isolation procedure (HP); (2) deionized water isolation procedure (WP); and (3) sodium hydroxide isolation procedure (NP) for djulis starch.

HP, WP, and NP starch exhibited the normal pasting feature of cereal and roots starch (Figure 4). Nevertheless, Table 4 shows that the significant differences were marked in pasting parameters. NP treatment starch indicated the lowest pasting viscosity because alkaline treatment can damage the structure of starch irreversibly and lead to a reduction in the pasting viscosity. This result agreed with previous studies, with lower peak viscosity with the alkaline treatment when compared with enzymatic treatment [33], though viscosity also followed the same trend. Breakdown viscosity in starch was lowest with HP, but highest with WP. Final viscosity was highest with WP at 5462.33 cP, followed by HP and NP at 5113.17 and 5104.73 cP, respectively. The increase in the final viscosity might be due to the aggregation of amylose molecules.

The measurement of the degree of re-association during cooling between the starch molecules concerning the amylose that leached from the swollen starch granules is called the setback viscosity. It is ordinarily practiced as a benchmark of the gelling ability or retrogradation tendency of starch [27]. Setback viscosity is obviously concerned with the starch isolation method used, which showed smaller values for the HP isolation treatment. The low setback viscosity (1139.01 cP) can be associated with a reduction in amylose leaching; the presence of granule fragments, which are due to a low breakdown viscosity; and a lower proportion of long amylopectin chains [13].

The pasting temperatures of HP, WP, and NP isolated starch were 71.70 °C, 72.80 °C, and 69.53 °C, respectively, which were accurate to the gelatinization temperature acquired by DSC analysis. Thus, djulis starch isolated by NP may exhibit a low pasting temperature, but high consistency, and stability, which is an interesting feature, which is suitable for foodstuff and commodities that involve high viscosity at a lower processing temperature.

## 3. Conclusions

Djulis starch isolated in this study were expressively different in terms of their physicochemical and functional properties. The three different starch isolation procedures used, HP, WP, and NP, produced high recovery starch with a low amount of damage caused. Generally, djulis starch isolated by the three procedures pretend to have an analogous morphological structure. The djulis starch granules were polygonal and angular in shape; the granule size was between 1.57 and 1.84, presenting a Maltese cross. Djulis starch can be classified as A-type, with a high relative crystallinity value (>30%), and has similar interaction between starch molecules. In conclusion, the NP isolation procedure with a steady and substantial reaction is fascinating from a technological point of view.

Djulis starch had a lower swelling power, pasting, and gelatinization temperature, but higher values for amylose content and pasting viscosity when compared with the A-type x-ray diffraction pattern of cereal starch. The analysis of various properties of djulis starch by the different isolation procedures provides beneficial information accompanied by the functional properties for use in various food industries. Djulis starch might be favorable for enhancing the texture of pasta noodles, for example, as a gelling agent applied to high viscous foods, the replacement of chemical modified starches used in baking and other formulation foods as well as a fat substitute and a binder with oral active ingredients. Moreover, due to the small granule size, djulis starch might have broad utilization as an edible biodegradable film. The fascinating and exclusive functional peculiarity of djulis starch could advantageously be applied in the manufacture of specialty food production. Further analysis toward the chemical composition and bioactive components such as the phenolic and flavonoid compositions of djulis hulls, roots, and starch by high performance liquid chromatography (HPLC) is required for the development of djulis functional enriched foods.

## 4. Experimental

### 4.1. Starch Isolation Procedure

Fresh grains of djulis were cultivated on November 2018 at a local farm (Pingtung, Taiwan). The grains of djulis were indiscriminately harvested at a mature stage. All samples were transferred into transparent low-density polyethylene (LDPE) zipper bags to prevent any external contamination and stored at a cooler box to transfer back to the laboratory.

**Figure 5:**
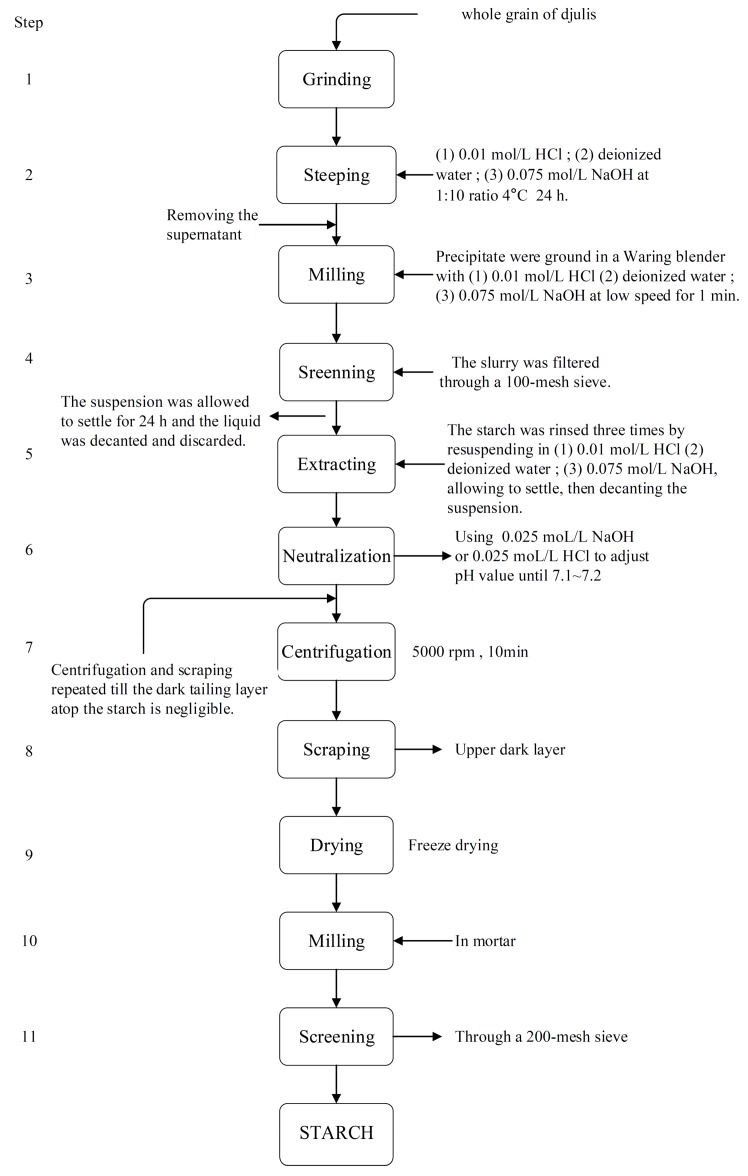
(1) Hydrochloric acid (HP); (2) deionized water (WP); and (3) alkali (NP) isolation procedures for djulis starch.

Starch was detached from the djulis grain flours by the subsequent three procedures of (1) the hydrochloric acid (HCl) isolation procedure (HP); (2) deionized water isolation procedure (WP); and (3) alkali (sodium hydroxide, NaOH) isolation procedure (NP). Starch was separated from djulis flour as described [14,34,35] with some modifications. The ground whole grain of djulis flour was steeped with (1) 0.01 mol/L HCl (HP), (2) deionized water (WP), and (3) 0.01 mol/L NaOH (NP), with the ratio of 1:10 at 4 °C for 24 h. After centrifugation, the supernatant was removed, while the precipitates were ground in a waring blender with (1) 0.01 mol/L HCl (HP), (2) deionized water (WP), and (3) 0.01 mol/L NaOH (NP) at lower speed for 1 min. The slurry was filtered through a 100-mesh sieve and the suspension was allowed to settle for 24 h and the liquid was decanted and discarded. The starch was rinsed three times by resuspending in (1) 0.01 mol/L HCl (HP), (2) deionized water (WP), and (3) 0.01 mol/L NaOH (NP), allowed to settle, followed by decanting the suspension. The djulis sample was neutralized by using 0.025 mol/L HCl or 0.025 mol/L NaOH to adjust the pH value to 7.1–7.2. Centrifugation and scraping was repeated until the dark tailing layer atop the starch was negligible. Next, the upper dark layer was scraped and freeze dried to obtain the djulis starch sample (Figure 5).

### 4.2. The Chemical Composition of Starch

The moisture (934.01), protein (984.13), lipid (954.02), and ash (942.05) content of the isolated djulis starch was determined according to Association of Official Analytical Chemists (AOAC) methods. The dry matter content was obtained by subtracting the moisture content from the total weight of the sample.

### 4.3. Starch and Amylose Content

The starch content of djulis starch was determined by the American Association of Cereal Chemists (AACC) methods (1995), which is by using the Megazyme total starch content assay kit (Megazyme International Ireland Ltd., Wicklow, Ireland). The amylose content of djulis starch was examined by the iodine adsorption technique [12].

### 4.4. Production Yield and Recovery

The yield and recovery of djulis starch obtained by HP, WP, and NP were calculated according to Equations (1) and (2) below:

(1) $$\mathrm{Yield}\;\left(\%\right)=\frac{Starch\;isolated\;\left(g\right)}{Initial\;sample\;weight\;\left(g\right)}\times 100$$

(2) $$\mathrm{Recovery}\;\left(\%\right)=\frac{Starch\;isolated\;\left(g\right)}{Total\;starch\;content\;\left(g\right)}\times 100$$

### 4.5. Granule Size

The granule size of starch was measured by using a laser particle sizer (Analysette 22 compact Laser Particle Sizer, Fritsch, Germany). The starch samples were dissolved in deionized water at a concentration of 4%. The device output had the volume spreading of starch granules as the essential measurement. The output data D ‘4, 3’ indicate that the granule diameter resulted from the volume spreading.

### 4.6. Morphological Observation of Starch Granule

Scanning electron microscopy (SEM) involved the use of the instrument with a model ABT-150S system (Topon Corp., Kyoto, Japan). Double-sided adhesive tape was used to attach the isolated starch on an aluminum stub. The sample was coated with gold-palladium (Model JBS-ES 150, Ion sputter coater, Topon Corp., Japan). In addition, 15 kV of accelerating potential was used during SEM.

For morphological analysis, a starch suspension (1% w/w) was ready with the addition of 50% glycerol. A drop of a starch suspension was placed on a slide and enclosed with a coverslip. An Olympus BX53 polarized light microscope (PLM) equipped with a CCD camera was used to observe the starch’s granule shape and Maltese cross.

### 4.7. X-Ray Diffraction

X-ray diffraction of the starch samples was evaluated using a Siemens x-ray diffractometer (Model D5000, Siemens Co., Madison, WI, USA) with the conducting requirement of 45 kV of target voltage, 30 mA of current, (2*θ*) 5–40° scanning range, 0.02 °/s scan speed, and 0.2 nm receiving slit width. The percentage crystallinity was calculated using Equation (3) as follows:

(3) $$\mathrm{Crystallinity}\;\left(\%\right)=\frac{Area\;under\;peaks}{Total\;area}\times 100$$

### 4.8. Swelling Power

The swelling power or swelling index of starch was determined as described by Waliszewski et al. [36] with modifications. Starch suspension (1% w/w) was arranged in flasks and heated up to the temperature of 60 °C, 70 °C, 80 °C, and 90 °C, respectively for 30 min with shaking every 5 min. Samples were left to cool to room temperature, then centrifuged at 4000 g for 15 min. The supernatant was removed, while the precipitates were dried and weighed for further calculation of the swelling power.

### 4.9. Thermal Properties

Thermal properties of the starch samples isolated by the three different treatments were analyzed by testing with a differential scanning calorimeter (Model DSC822, Mettler-Toledo Co., Greifensee, Switzerland). A total of 3 mg of starch samples and 9 mg of deionized water were loaded into 40 μL aluminum differential scanning calorimeter (DSC) crucibles that were hermetically sealed. The sealed crucibles were left overnight at room temperature before DSC analysis to acquire an equal spreading of water before heating the calorimeter. An unoccupied aluminum DSC crucible was used as a reference. The scanning temperature series was increased from 40 to 100 °C at a heating rate of 5 °C/min. The onset (T_0_), peak (T_p_) and concluding (T_c_) gelatinization temperatures, enthalpy (ΔH) of gelatinization, and gelatinization range of dry starch were calculated automatically.

### 4.10. Pasting Properties

A Rapid Visco Analyzer (Model RVA-3D, Newport Scientific, Sydney, Australia) was used to investigate the pasting properties of isolated starch. Starch slurries containing 8% w/w starch (28 g of total weight) were readied, then subjugated to a heating–cooling cycle. The starch slurries were equilibrated at 50 °C for 1 min, heated up to 95 °C at 6 °C/min, retained at a temperature at 95 °C for 5 min, then cooled to 50 °C at 6 °C/min, and then the temperature controlled at 50 °C for 2 min. The paddle speed was fixed at 960 rpm for the first 10 s of the experiment, followed by 160 rpm for the remainder of the experiment. The viscoamylograph elucidated the various features of the starch as well as the peak temperature, pasting temperature, peak viscosity, viscosity at 95 °C after 5 min holding, and the final viscosity at the end of the 50 °C holding period, which was accessed from a typical diagram of the Rapid Visco Analyzer pasting curves [37]. Each sample was carried out with three replications.

### 4.11. Statistical Analysis

The mean (SD) of each analysis are reported. In the statistical analysis of this experiments, all data were evaluated by using a single-factor of ANOVA. If the F-value was significant (*p* < 0.05) on ANOVA, then a Duncan’s new multiple range test was used to correlate the treatment means.

## Figures and Tables

**Table 1:** Chemical composition of djulis starch by the three treatment procedures.

Process	Moisture (%)	Protein (%) *	Lipid (%) *	Ash (%) *	Amylose (%)	Damage Starch (%)
HP	4.89 ± 0.21 ^c^	0.29 ± 0.02 ^b^	0.82 ± 0.07 ^b^	0.11 ± 0.02 ^a^	22.14 ± 0.31 ^b^	3.93 ± 0.27 ^a^
WP	6.35 ± 0.36 ^a^	0.37 ± 0.07 ^a^	0.94 ± 0.04 ^a^	0.12 ± 0.01 ^a^	24.15 ± 0.28 ^a^	2.12 ± 0.19 ^c^
NP	5.48 ± 0.19 ^b^	0.22 ± 0.03 ^b^	0.75 ± 0.05 ^b^	0.14 ± 0.03 ^a^	22.43 ± 0.26 ^b^	3.05 ± 0.11 ^b^

HP, hydrochloric acid (HCl) isolation procedure; WP, deionized water isolation procedure; NP, alkali (NaOH) isolation procedure. Data are means ± SD. Means with the same letters in a column do not differ significantly (*p* < 0.05) (*n* = 3). * Dry weight basis.

**Table 2:** Yield, recovery, and granule size of djulis starch by the three treatment procedures.

Process	Yield (%)	Recovery (%)	Starch Granule Size	
			Range (μm)	Mean (μm)
HP	29.74 ± 0.45 ^a^	78.49 ± 0.76 ^a^	0.56−1.96	1.57 ± 0.09 ^b^
WP	30.19 ± 0.29 ^a^	79.68 ± 0.58 ^a^	0.74−3.02	1.84 ± 0.13 ^a^
NP	26.41 ± 0.33 ^b^	69.15 ± 0.26 ^b^	0.62−2.48	1.62 ± 0.21 ^b^

Data are means ± SD. Means with the same letters in a column do not differ significantly (*p* < 0.05) (*n* = 3).

**Table 3:** Gelatinization properties of djulis starch by the three treatment procedures.

Process	T_0_ (°C)	T_p_ (°C)	T_c_ (°C)	ΔT(°C)	ΔH (J/g)
HP	59.45 ± 0.47 ^a^	64.35 ± 0.62 ^a^	72.19 ± 1.73 ^a^	12.74 ± 0.44 ^b^	9.24 ± 1.03 ^a^
WP	60.27 ± 0.54 ^a^	65.21 ± 0.22 ^a^	72.95 ± 0.38 ^a^	12.68 ± 0.31 ^b^	8.51 ± 0.68 ^a^
NP	60.73 ± 0.89 ^a^	66.02 ± 1.10 ^a^	70.74 ± 1.35 ^a^	10.01 ± 0.25 ^a^	6.95 ± 0.98 ^b^

Data are mean ± SD. Means with the same letters in a column do not differ significantly (*p* < 0.05) (*n* = 3). T_0_, onset gelatinization temperature; T_p_, peak gelatinization temperature; T_c_, concluding gelatinization temperature; ΔT, gelatinization temperature range (ΔT = T_c_ − T_0_); ΔH, enthalpy of gelatinization.

**Table 4:** Pasting characteristics of djulis starch by the three treatment procedures.

Process	PV (cP)	TV (cP)	BD (cP)	FV (cP)	SB (cP)	PT (°C)
HP	4674.67 ± 61.45 ^b^	3974.33 ± 33.71 ^a^	772.67 ± 167.05 ^c^	5113.17 ± 16.56 ^b^	1139.00 ± 50.27 ^c^	71.70 ± 0.30 ^a^
WP	5200.01 ± 22.61 ^a^	3683.67 ± 58.71 ^b^	1516.33 ± 37.07 ^a^	5462.33 ± 28.50 ^a^	1778.67 ± 51.39 ^b^	72.80 ± 0.61 ^a^
NP	4397.67 ± 17.02 ^c^	3248.00 ± 83.29 ^c^	1149.67 ± 90.31 ^b^	5104.73 ± 59.72 ^b^	1856.33 ± 37.31 ^a^	69.53 ± 0.29 ^b^

Data are means ± SD. Means with the same letters in a column do not differ significantly (*p* < 0.05) (*n* = 3). Different letters within columns present significant differences (*p* < 0.05). PV, peak viscosity; TV, through viscosity; BD, breakdown viscosity; FV, final viscosity; SB, setback viscosity; PT, pasting temperature.
